# RETRACTED: Rehman et al. Nicotinamide Ameliorates Amyloid Beta-Induced Oxidative Stress-Mediated Neuroinflammation and Neurodegeneration in Adult Mouse Brain. *Biomedicines* 2021, *9*, 408

**DOI:** 10.3390/biomedicines14030639

**Published:** 2026-03-12

**Authors:** Inayat Ur Rehman, Riaz Ahmad, Ibrahim Khan, Hyeon Jin Lee, Jungsung Park, Rahat Ullah, Myeong Jun Choi, Hee Young Kang, Myeong Ok Kim

**Affiliations:** 1 Division of Life Sciences and Applied Life Science (BK 21 Four), College of Natural Science, Gyeongsang National University, Jinju 52828, Republic of Korea; inayaturrehman201516@gnu.ac.kr (I.U.R.); riazk0499@gnu.ac.kr (R.A.); ibrahim1994@gnu.ac.kr (I.K.); lhj4912@gnu.ac.kr (H.J.L.); jsp@gnu.ac.kr (J.P.); rahatullah1414@gnu.ac.kr (R.U.); 2 Research and Development Center, Axceso Bio-Pharma Co., Ltd., Anyang 14056, Republic of Korea; myeongjun@gmail.com; 3 Department of Neurology, Gyeongsang National University Hospital, Gyeongsang National University, College of Medicine, Jinju 52828, Republic of Korea; miranda75@naver.com

The journal retracts the article titled “Nicotinamide Ameliorates Amyloid Beta-Induced Oxidative Stress-Mediated Neuroinflammation and Neurodegeneration in Adult Mouse Brain” [1], cited above.

Following publication, concerns were brought to the attention of the Editorial Office regarding the integrity of figures presented in this publication [1].

In accordance with the journal’s standard procedures, the Editorial Office and Editorial Board conducted an investigation that identified indications of inappropriate editing and partial duplication between Figure 2D,E and Figure 4B [1], presenting different experimental conditions. While the authors collaborated with this process, raw material meeting the journal’s requirements for original images could not be provided for Editorial evaluation. Consequently, the Editorial Board has lost confidence in the reliability of the findings and has decided to retract this publication, as per MDPI’s retraction policy.

This retraction was approved by the Editor-in-Chief of the *Biomedicines* journal. 

The authors have been informed of this retraction but did not provide a comment on this decision.
